# Dietary Fiber’s Physicochemical Properties and Gut Bacterial Dysbiosis Determine Fiber Metabolism in the Gut

**DOI:** 10.3390/nu16152446

**Published:** 2024-07-27

**Authors:** Edward Moncada, Nuseybe Bulut, Shiyu Li, Timothy Johnson, Bruce Hamaker, Lavanya Reddivari

**Affiliations:** 1 Department of Food Science, Purdue University, West Lafayette, IN 47907, USA; emoncada@purdue.edu (E.M.); nbulut@purdue.edu (N.B.); lishiyu5211@126.com (S.L.); hamakerb@purdue.edu (B.H.); 2 Department of Animal Science, Purdue University, West Lafayette, IN 47907, USA; john2185@purdue.edu

**Keywords:** dietary fiber, fiber fermentation, gut bacterial dysbiosis, colitis

## Abstract

A fiber-rich diet is considered beneficial for gut health. An inflamed gut with a dysbiotic bacterial community can result in altered fiber metabolism depending on the fiber’s physicochemical properties. This study examined the effect of fiber’s physicochemical properties on fiber fermentation in the presence of healthy and colitis-associated bacteria. Sixteen fibers with different levels of solubility, complexity, and fermentation rate were used in in vitro fermentation with healthy human gut bacteria. Resistant maltodextrins (RMD), pectin (HMP), inulin (ChIn), and wheat bran (WB) were selected for fermentation using ulcerative colitis (UC)-associated bacteria to assess bacterial dysbiosis effect. UC-associated gut microbiota showed a significant reduction in α-and β-diversity indices compared to healthy-associated microbiota. The differences in the gut microbiota composition and diversity between the donors resulted in decreased fermentation rates with UC-associated bacteria. Fiber fermentation metabolites, short-chain fatty acids (SCFA) and gas production were significantly lower in the presence of UC-associated bacteria for all four fibers tested. Overall, we conclude that dietary fiber properties and microbial dysbiosis are influential in fiber fermentation and metabolite production in the gut.

## 1. Introduction

The prevalence of chronic inflammation and related diseases/disorders, including inflammatory bowel disease (IBD), are on the rise in the US [1]. Altered gut microbiome and barrier dysfunction play a vital role in the development of UC, a form of IBD [2,3,4]. Accumulating evidence suggests that dietary fibers have the potential to exert health benefits, such as protection against intestinal mucus degradation, inflammation, and gut barrier dysfunction, through gut bacteria modulation and SCFA production [5,6]. Despite their potential health benefits, colitis patients report poor tolerance to dietary fiber [7]. Even though low-fiber diets have been associated with a greater risk of IBD and cardiovascular diseases, colitis patients are usually advised not to consume fiber-rich foods during flare-ups. The question is whether UC-associated fiber intolerance is independent of the type of fiber. Fiber intolerance would refer to the discomfort fiber consumption can provoke, such as bloating, flatulence, diarrhea, amongst others. Specifically in a UC context, it would refer to the exacerbation of inflammatory conditions in the colon. The alleviation or worsening of inflammatory symptoms could be associated with the physicochemical characteristics of the fibers. Physiological effects are also dependent on dietary fiber’s physicochemical properties, which determine their metabolism and fermentation by the gut bacteria [8]. Fiber solubility has been shown to play a role in stool consistency [9]. Moreover, soluble fibers are assumed to be associated with a more rapid fermentation rate [10]. The fermentation rate of dietary fiber, which is determined by its structure, solubility, viscosity, and accessibility [11], is vital due to its relationship with gas production. Higher gas production is associated with intolerance due to bloating, stomach distension and increased flatulence [12,13]. The structural complexity of fiber, such as branching, linkage types, chain length, degree of polymerization, molecular weight, particle size and even source [14,15], influences bacterial capacity to physically access the fibers. Subtle variations in dietary fibers’ structure may favor bacteria [14] that may be beneficial for gut health, such as butyrate producers in the competitive colon environment. In addition, a fiber with coarse particle size has been found to provoke discomfort by exacerbating the inflammation in the walls of the intestine [16].

Though dietary fibers play a significant role in gut health [17], a critical gap remains in our understanding of the diverse response of dietary fibers with respect to physicochemical properties and gut health, making fiber selection for UC symptoms difficult. Understanding the influence of fiber’s physicochemical properties and gut microbial dysbiosis in fiber fermentation and metabolism is important for selecting well-tolerated fibers, as gut bacterial dysbiosis is one of the major hallmarks of UC patients. We hypothesized that UC-associated bacterial dysbiosis influences the metabolism of fibers, and fibers will differ in their tolerance levels based on their physicochemical properties.

## 2. Materials and Methods

### 2.1. Experimental Design

**Figure 1:**
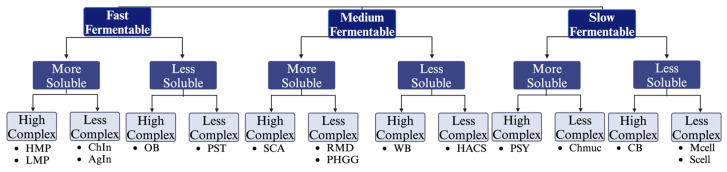
Physicochemical properties used to select and classify dietary fibers for this study. High Methoxyl Pectin (HMP), Low Methoxyl Pectin (LMP), Inulin from Chicory (ChIn), Inulin from Agave (AgIn), Oat Bran (OB), Raw Potato Starch (PST), Soluble Corn Arabinoxylans (SCA), Resistant Maltodextrins (RMD), Partially Hydrolyzed Guar Gum (PHGG), Wheat Bran (WB), High Amylose Corn Starch (HACS), Psyllium Husk (PSY), Chia Mucilage (Chmuc), Corn Bran (CB), Medium sized cellulose (Mcell), Small sized cellulose (Scell).

Dietary fibers were classified based on physicochemical properties, including fermentability rate, solubility, and complexity (Figure 1). Fibers were initially assigned to these categories based on the existing literature.

### 2.2. Dietary Fibers

As listed in Table 1, sixteen fibers were bought commercially from different suppliers, extracted, or donated by collaborating laboratories.

### 2.3. Solubility Measurements of Dietary Fibers

The AOAC 991.43 method was applied using the Total Dietary Fiber Assay Kit (K-TDFR from Megazyme, Lansing, MI, USA), to determine the soluble (SDF) and insoluble (IDF) fractions of each of the fibers tested. The total dietary fiber (TDF) was determined as a sum of the SDF and IDF. The AOAC 991.43 method was developed for the TDF quantification of high molecular weight dietary fibers; we used this method to quantify soluble fractions in 3 low molecular weight fibers (ChIn, AgIn and RMD). We are aware that the use of this method may be underestimating the SDF fraction of the low molecular weight fibers.

### 2.4. In Vitro Upper Gastrointestinal Digestion

Before in vitro microbial fermentation, fibers were subjected to upper gastrointestinal digestion according to INFOGEST protocol with minor modifications [19]. Electrolyte solutions used to simulate the salivary fluid (SSF), gastric fluid (SGF), and intestinal fluid (SIF) were prepared following the details in the INFOGEST protocol. All steps were carried out at 37 °C and under continuous agitation on a magnetic stirrer. In the oral digestion phase, 15 g of sample, 24 mL of SSF, and 2 mL of the salivary amylase mixture was added and incubated for 5 min; pH was continuously monitored to be at 7. The saliva-digested sample was then mixed with 24 mL of pre-heated SGF to simulate the gastric phase. pH was adjusted to 2.0 with HCl before adding 6 mL of porcine pepsin and 15 μL of CaCl_2_ to the mixture and incubated for 2 h. The pH of the gastric mixture was monitored to be between 2.0 and 3.0 during this incubation period. In the intestinal digestion phase, 12 mL of pre-heated SIF electrolyte solution was added to the gastric digested sample and pH was adjusted to 7.0 with NaOH (1 M). Pancreatin and bovine bile salts were added to reach concentrations of 100 U/mL and 10 mM, respectively. Then, 600 μL of CaCl_2_ was added to the mixture and incubated for 2 h, while monitoring pH to be 7.0. The digested sample was then heated to 80 °C to terminate the action of any enzyme. Samples were dialyzed against distilled water for 48 h with two changes of water and then freeze-dried and stored at −80 °C for further use.

### 2.5. In Vitro Human Fecal Fermentation

Digested substrates were used for in vitro human fecal fermentation, performed as previously described [20] with few minor modifications. The total carbohydrate amount in the samples was determined by the phenol-sulfuric acid method [21]. A 50 mg equivalent of carbohydrates was weighed in triplicate test tubes for each time point designated for the batch. A carbonate–phosphate buffer was prepared and autoclaved at 121 °C for 20 min. The buffer was cooled down and saturated by adding cysteine hydrochloride solution (0.1 g/mL). The carbonate–phosphate buffer was placed inside an anaerobic chamber 48 h before performing the fermentation to remove all oxygen present. Test tubes with samples were also placed inside the chamber 24 h before the fermentation and hydrated with 4 mL of the buffer. Healthy human fecal samples were obtained from two healthy volunteers who had not taken antibiotics in the last 3 months according to the IRB protocol (IRB-2020-7). Healthy donors were also age and sex-matched (males, 25–35 years of age). A fecal sample from a single adolescent male UC patient was collected in the Seattle Children’s Hospital, shipped, and kept at −80 °C until its use. The UC donor had a fecal calprotectin level of 1947 μg/mg; this was quantified to assess for disease severity. No significant differences were observed for the inoculum from healthy and UC donors for initial bacterial counts. Under anaerobic conditions, fecal samples were pooled, homogenized with the carbonate–phosphate buffer with a ratio of feces to buffer (1:3, *w*/*v*) and filtered through 3 layers of cheesecloth. Samples in each test tube were inoculated with 1 mL of the fecal slurry. Tubes without any added carbohydrates were used as negative controls for each time point. Tubes were then sealed and incubated at 37 °C in a shaking incubator. Tubes were removed at different time points (0, 6, 12, 24 and 48 h) of fermentation from the incubator. Total gas volume was measured using graduated syringe displacement. Tubes were then unsealed to terminate microbial activity, and the pH of samples was recorded. For SCFAs, aliquots of 400 μL were taken, and for bacterial sequencing analysis samples were precipitated and a pellet was used. Both samples were kept at −80 °C for further analysis.

### 2.6. Short-Chain Fatty Acid Quantification

Before storing aliquots at −80 °C for SCFA analysis, 100 μL of a mixture containing 50 mM 4-methyl-valeric acid (No. 277827-5G, Sigma-Aldrich Inc., St. Louis, MO, USA), 5% meta-phosphoric acid, and copper sulfate (1.56 mg/mL) was added and vortexed. For quantification, samples were thawed, vortexed and centrifuged at 15,000× g for 10 min. An aliquot of 0.3 μL of the supernatant fraction was injected into a GC-FID 7890A (Agilent Technologies, Inc., Santa Clara, CA, USA) equipped with a fused silica capillary column (NukolTM, Supelco No. 40369-03A, Bellefonte, PA, USA). The initial oven temperature was held at 50 °C for 2 min, ramped to 70 °C at a rate of 10 °C/min, to 85 °C at a rate of 3 °C/min, to 110 °C at a rate of 5 °C/min, to 290 °C at a rate of 30 °C/min, and finally held at 290 °C for 8 min. Helium was used as a carrier gas at a constant flow rate of 1 mL/min.

### 2.7. Bacterial Sequencing

For the microbiome analysis, metagenomic DNA was extracted using the QIAmp PowerFecal Pro DNA Kit (QIAgen, Germantown, MD, USA) according to the manufacturer’s instructions. To analyze the bacterial communities, samples were sent to the Department of Physiology and Pharmacology in the University of Toledo (University of Toledo, Toledo, OH, USA), in which the hypervariable 16S V3-V4 region was amplified and sequenced using the Illumina MiSeq Platform (Illumina, Inc., San Diego, CA, USA). Library preparation and 16S rRNA gene sequencing were also performed at the same facility. Bioinformatic analysis was conducted using the QIIME2 (v.2-2021.2) and R software (version 2021.09.2). Using the DADA2 [22] denoising step, the forward and reverse sequences were both trimmed at position 60, and the forward and reverse sequences were truncated at position 271 and 280, respectively. The forward and reverse reads were then merged. Taxonomy was assigned using SILVA 138 region database [23]. Sequences were then rarefied to 53,500 sequences per sample to calculate the alpha and beta microbial diversity; samples with low sequence counts were removed in this step. Alpha diversity was estimated in QIIME2 using Faith (Faith’s PD) for phylogenetic diversity [24] and the Shannon Index for richness and evenness. The Bray–Curtis Dissimilarity Index and Weighted UniFrac were used to analyze the Beta diversity [25]. The 20 most relatively abundant bacteria at the genus level were stacked in a bar graph to show the differences between donors and treatments.

### 2.8. Statistical Analysis

Data were analyzed and graphed in R software and GraphPad prism 10. Outliers were identified using the robust regression and outlier removal (ROUT) method. Data sets were analyzed for normality using the Shapiro-Walk test, and if variables passed the test then they were analyzed utilizing *t*-test, one-way and two-way ANOVA, Fisher’s LSD multiple comparison test and Pearson correlation test. For variables not following normality, a Mann–Whitney or a Kruskal–Wallis test followed by Dunn’s multiple comparison test was used. For bacterial sequencing analysis, statistical differences in beta diversity were tested using permutational multivariate analysis of variance (PERMANOVA) in QIIME2. Permutational multivariate analysis of dispersions (PERMDISP) was also performed in QIIME2 to determine significant differences in group variances. Data are presented as the mean ± standard error of the mean (SEM). *p*-values below 0.05 were considered statistically significant. Statistical significance is indicated as follows: ns = no significance, * = *p* < 0.05; ** = *p* < 0.01; *** = *p* < 0.001, **** = *p* < 0.0001.

## 3. Results

### 3.1. Dietary Fiber Solubility Measurements

Quantification of the soluble and insoluble fractions served to classify the dietary fibers in their respective category (more or less soluble). As observed in Table 2, fibers with a SDF fraction higher than 50% were categorized as more soluble, while the ones with more than 50% of the IDF fraction were categorized as less soluble fibers.

### 3.2. In Vitro Fermentation of Dietary Fibers Inoculated with a Healthy Human Fecal Sample

**Figure 2:**
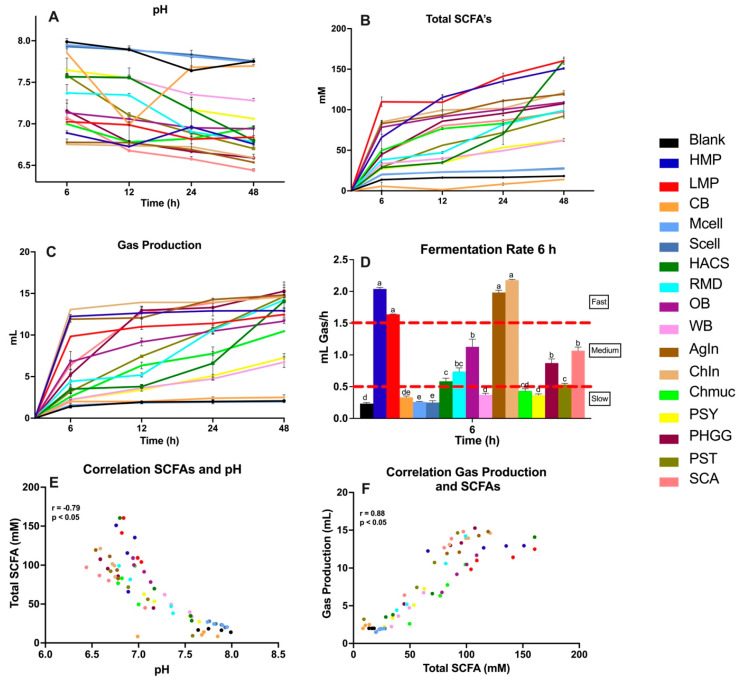
pH variations (**A**), total SCFA (**B**), and gas production (**C**) during the in vitro healthy human fecal fermentation of the sixteen selected fibers. Fermentation rate (**D**) was determined within the 6 h time point. Correlation analysis between total SCFAs and pH (**E**) and between gas production and total SCFAs (**F**). Blank was used as a negative control. Triplicates of each fiber (*n* = 3) for every time point are shown in the figure. Different letters between bars indicate significant differences (*p* < 0.05).

Dietary fibers were subjected to in vitro upper GI digestion before in vitro fecal fermentation using the sample from a healthy donor. pH, gas production and SCFAs were analyzed at 6, 12, 24 and 48 h of fermentation. During fermentation, pH generally reduced with time, irrespective of the type of fiber (Figure 2A). Fibers that showed a lower pH by 48 h are known to be highly fermentable, the most noticeable ones being both the Chicory (ChIn) and Agave Inulin (AgIn), classified as having a simple structure, being fast fermentable and highly accessible to different bacteria. The fermentation of pectin resulted in a higher production of SCFAs, followed by both inulins compared to other fibers throughout all the time points (Figure 2B), except for the high amylose corn starch (HACS), which resulted in a noticeable amount of total SCFAs between 24 and 48 h. A similar trend was observed in gas production (Figure 2C), where both pectins and inulins showed higher gas production at 6 h and maintained their production in the following hours. The fermentation rate (mL Gas/h) was calculated by dividing the amount of gas produced during the first 6 h of fermentation with time. Dietary fibers that produced more than 1.5 mL of gas/h were considered fast fermentable fibers, while fibers that produced between 0.5 and 1.5 mL of gas/h were classified as medium fermentable, and fibers below the 0.5 mL of gas/h rate were considered as slow (Figure 2D). This sorting helped us confirm the initial classification of fibers. A negative correlation (r = −0.79) was observed between the production of total SCFAs and pH (Figure 2E), meaning that higher production of total SCFAs reduced the intestinal pH. Additionally, a positive correlation (r = 0.88) was observed between gas and total SCFA production (Figure 2F).

**Figure 3:**
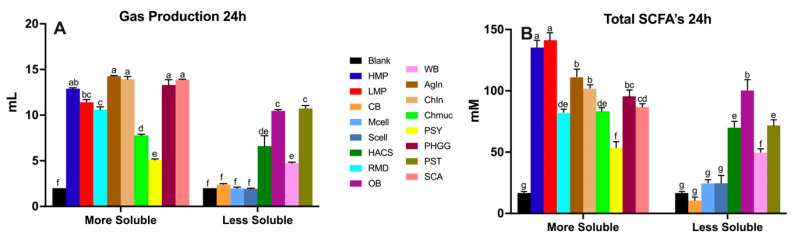
Comparison of gas production (**A**) and total SCFA (**B**) production at the 24 h time point during in vitro fecal fermentation depending on fiber solubility. Blank was used as a negative control. Triplicates of each fiber (*n* = 3) for every time point are shown in the figure. Different letters between bars indicate significant differences (*p* < 0.05).

Most soluble fibers had higher total SCFA and gas production (Figure 3). Certain soluble fibers such as chia mucilage (Chmuc) and psyllium husk (PSY) were less fermentable, and certain less soluble fibers such as oat bran (OB) and potato starch (PST) showed relatively high gas production at the 24 h time point. These less soluble fibers equaled the gas production levels of some of the highly soluble fibers. A similar pattern was observed for the total SCFA production (Figure 3B). OB, HACS and PST showed equal or higher total SCFA production compared to certain soluble fibers, suggesting solubility may not be a good indicator of fermentability.

Based on the results from the in vitro fermentation using a healthy human fecal sample as inoculum, fibers were assigned to three scenarios. Scenario 1: medium total SCFA production–high gas production, scenario 2: high total SCFA production–high gas production, scenario 3: low total SCFA production–low gas production. Due to a positive correlation (R = 0.92 and *p* < 0.05) between gas and total SCFA production (Figure 2F), no fibers tested were categorized into high total SCFA production–low gas production. The correlation between those fermentation by-products also indicated that in scenario 1, the relationship of low total SCFA and high gas had to be modified to a medium production level of total SCFA. Fibers with high gas production were considered less tolerable and fibers with high total SCFA were considered more beneficial, due to the potential health benefits of these metabolites on the human body. This fiber classification (Table 3) served as a screening tool to determine which fibers would be used to perform the in vitro fermentation with the UC patient fecal sample to assess the role of gut bacterial dysbiosis on fiber fermentation and metabolism.

RMD, HMP, ChIn, and WB were selected to proceed into the gut bacterial dysbiosis experimentation. These four fibers were selected, maintaining at least one dietary fiber from each of the scenarios, which possess distinct physicochemical properties and have industry relevance.

### 3.3. In Vitro Fermentation of Dietary Fibers Inoculated with UC Patient Fecal Sample-Role of Gut Bacterial Dysbiosis

**Figure 4:**
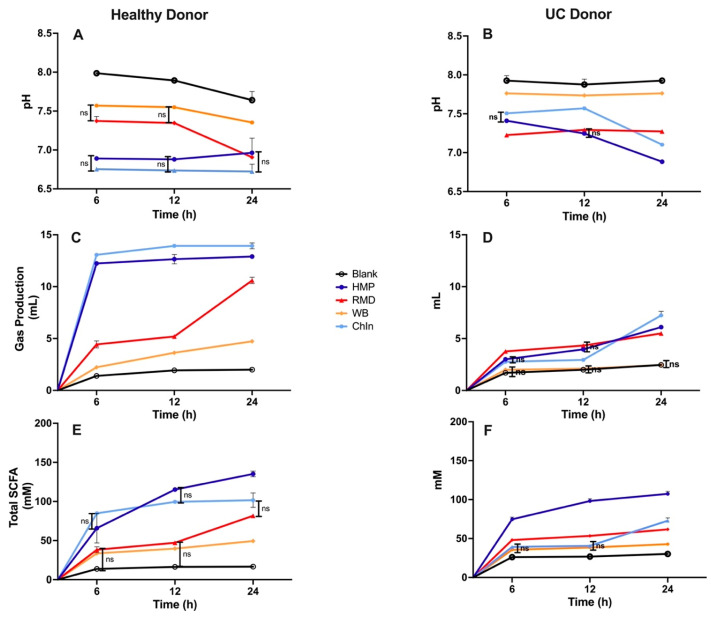
Comparing healthy vs. UC donor fiber fermentation parameters to assess the role of gut bacterial dysbiosis in fiber metabolism. Triplicates of each fiber (*n* = 3) for every time point are shown in the figure. (ns) means no significant difference; rest of the comparisons with no legend mean that significant differences were observed at *p* < 0.05. (**A**,**B**) pH measurements, (**C**,**D**) gas production and (**E**,**F**) total SCFA concentration at the distinct fermentation timepoints using (**A**,**C**,**E**) healthy donor bacteria and (**B**,**D**,**F**) UC donor bacteria as inoculum.

The selected four fibers were subjected to an in vitro fermentation using the UC patient fecal sample as inoculum. pH, gas production and total SCFA were measured and compared to the results obtained with the healthy inoculum fermentation. Here, we chose three time points (6, 12, 24 h). Fiber fermentation using healthy donor bacteria as inoculum generated pH differences between fiber types at the 6 h time point (Figure 4A). No significant differences were observed at 6 and 12 h time points between fast-fermentable fibers, ChIn and HMP, and between RMD and WB. A significant pH reduction at the 24 h time point was observed with RMD. Gas production was significantly different between all the dietary fibers throughout all fermentation time points (Figure 4C). A significantly higher production of total SCFAs was observed with highly fermentable fibers (ChIn and HMP) compared to the medium and slow fermentable fibers (RMD and WB) for time points 6 and 12 h (Figure 4E). At the 24 h time point, RMD fermentation resulted in a similar amount of total SCFAs compared to ChIn, which seemed to reach its plateau after the 12 h time point. HMP’s total SCFA concentration steadily increased with time from 0 to 24 h.

Fiber fermentation (as indicated by pH measurement) with the UC-associated bacteria generated no significant differences in pH between HMP and ChIn at the 6 h time point. RMD treatment showed the lowest pH at this time point (Figure 4B). pH was mostly constant throughout all the fermentation time points for RMD and WB. With time, HMP fermentation resulted in a significant reduction in pH. At the 24 h period, ChIn had a significant pH reduction, and the pairwise comparisons with other fibers were different (*p* < 0.05). In terms of gas production, we could observe that the fermentation of WB with UC-associated bacteria was limited, showing no significant differences compared to our blank through all fermentation periods (Figure 4D). HMP, RMD and ChIn all expressed different gas production levels at the 24 h period, with ChIn being the highest gas producer. Despite a higher amount of gas in the presence of ChIn, HMP fermentation resulted in an elevated total SCFA content throughout the fermentation time points (Figure 4F).

**Figure 5:**
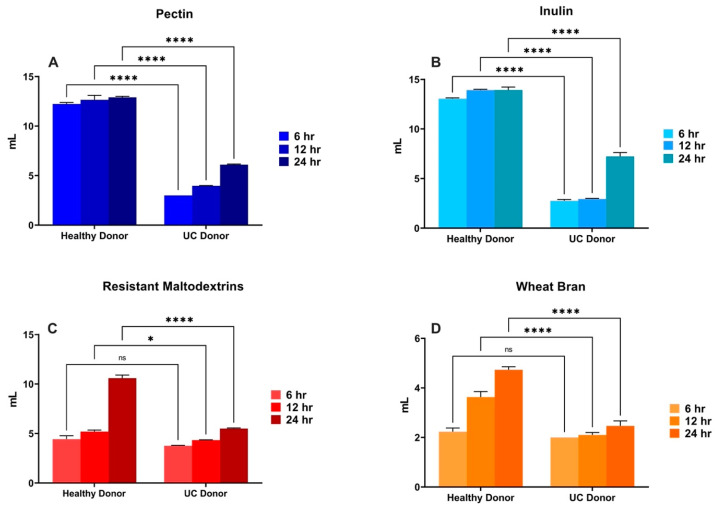
Comparison of healthy vs. UC donor effect on the gas production of each of the fiber treatments. Triplicates of each fiber (*n* = 3) for every time point are shown in the figure. An asterisk (*) and horizontal line represent a statistical difference (*p* ≤ 0.05) between the two groups. (****) represent *p* ≤ 0.0001 and (ns) means no statistical difference. Gas production differences at the distinct fermentation timepoints between the healthy and UC donor within the (**A**) HMP, (**B**) ChIn, (**C**) RMD, and (**D**) WB treatments.

**Figure 6:**
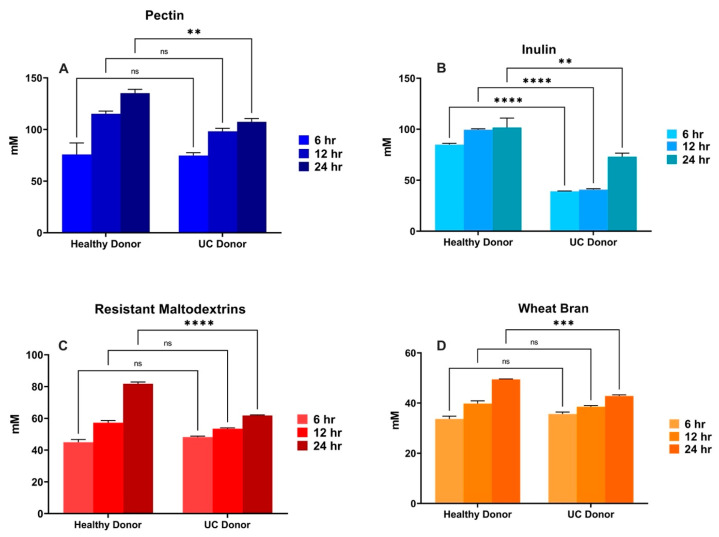
Comparison of healthy vs. UC donor effect on total SCFA production of each of the fiber treatments. Triplicates of each fiber (*n* = 3) for every time point are shown in the figure. Asterisks (**) and horizontal line represent a statistical difference (*p* ≤ 0.01) between the two groups. (***) represents *p* ≤ 0.001, (****) *p* ≤ 0.0001 and (ns) means no statistical difference. Total SCFA production differences at the distinct fermentation timepoints between the healthy and UC donor within the (**A**) HMP, (**B**) ChIn, (**C**) RMD, and (**D**) WB treatments.

Gas and total SCFA production differed between donors for each of the fibers tested (Figure 5 and Figure 6). We could observe that, for HMP (Figure 5A) and ChIn (Figure 5B) treatments, gas production at nearly all fermentation time points was significantly lower (*p* < 0.001) for the UC donor, indicating that these are the most affected fibers by bacterial dysbiosis. Furthermore, RMD (Figure 5C) and WB (Figure 5D) did not differ in their 6 h time point between donors, which could be associated with their medium–slow fermentation rate, while HMP and ChIn are fast-fermentable fibers, and their differences were noted earlier in the fermentation. For the ChIn treatment (Figure 6B), fiber fermentation with UC-associated bacteria significantly reduced (*p* < 0.001) the total SCFA production through all the time points. The same reduction effect was observed for the HMP, RMD and WB treatments, but only at the 24 h time point (Figure 6A,C,D).

### 3.4. Gut Bacterial Sequencing-Shifts during Healthy and UC Donor In Vitro Fermentation

**Figure 7:**
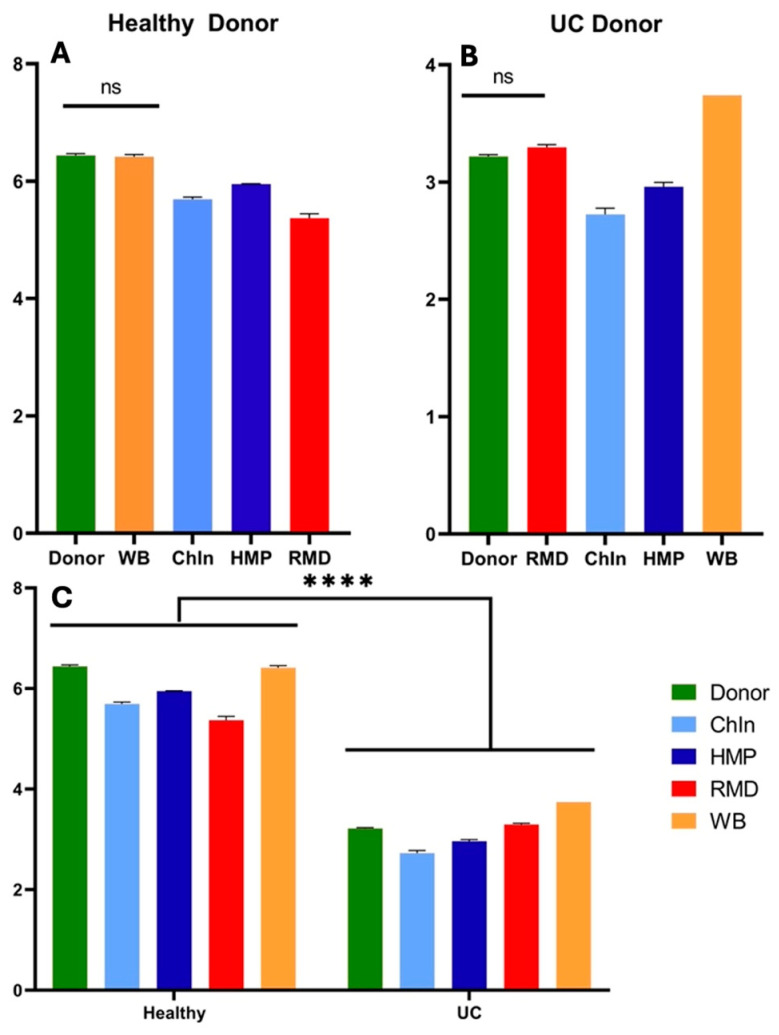
Alpha diversity measurements by Shannon Index of samples at 24 h fermentation as determined by 16S rRNA gene sequencing. Triplicates of each fiber (*n* = 3) were analyzed and expressed in the results. Asterisks (****) and horizontal line represent a statistical difference (*p* ≤ 0.0001) between the two groups. (ns) means no significant difference. Differences in Shannon indexes between fiber treatments and the initial donor fecal sample, (**A**) indexes within the healthy donor fermentation and (**B**) within the UC donor. (**C**) Overall comparison of alpha diversity values between the healthy and UC donors.

Bacterial community analysis resulted in WB being the only fiber that did not alter from the healthy donor’s initial Shannon Index value (Figure 7A). For the rest of the fiber treatments, alpha diversity was significantly different from the initial healthy donor sample. UC is driven by a shift in bacterial communities, especially in their richness and evenness. The Shannon Index, as a reference for richness and evenness, showed that the only fiber that did not differ within the UC donor was RMD. Interestingly, ChIn and HMP resulted in decreased diversity, while WB increased it. Significant differences were found between the donors, independent of the fiber (Figure 7C).

**Figure 8:**
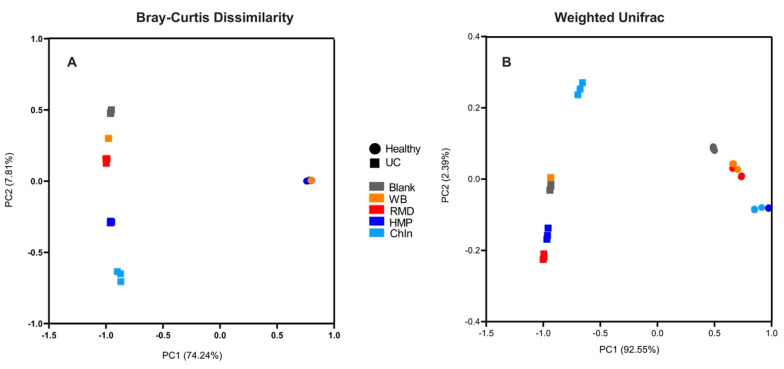
Principal coordinate analysis (PCoA) of Bray–Curtis dissimilarity (**A**) and Weighted UniFrac distances (**B**) between fiber treatments and donors. Triplicates of each fiber (*n* = 3) were analyzed.

To determine shifts in community structure (beta diversity), Bray–Curtis dissimilarity index (Figure 8A) and Weighted Unifrac (Figure 8B) were used. The PCoA plots show distinct clustering of healthy and UC donors by axis 1 (PC1), which explains 74.24% of the variance in the Bray–Curtis and 92.55% in the Weighted Unifrac. This can be attributed to the differences found between the donor communities. Dissimilarities between the fiber treatments within the donors can be explained by axis 2 (PC2), which accounted for only 7.81% for Bray–Curtis and 2.39% for Weighted Unifrac. Overall, both axes explained a total of 82.05% and 94.94% of the variations between the different bacterial communities for the Bray–Curtis and Weighted Unifrac, respectively. Analyses of the beta diversity also showed a common behavior of the samples in both measurements. Fiber treatments belonging to the healthy group are closely clustered with each other, while the variations observed between the fiber treatments in the axis were mostly due to UC-associated microbial dysbiosis.

**Figure 9:**
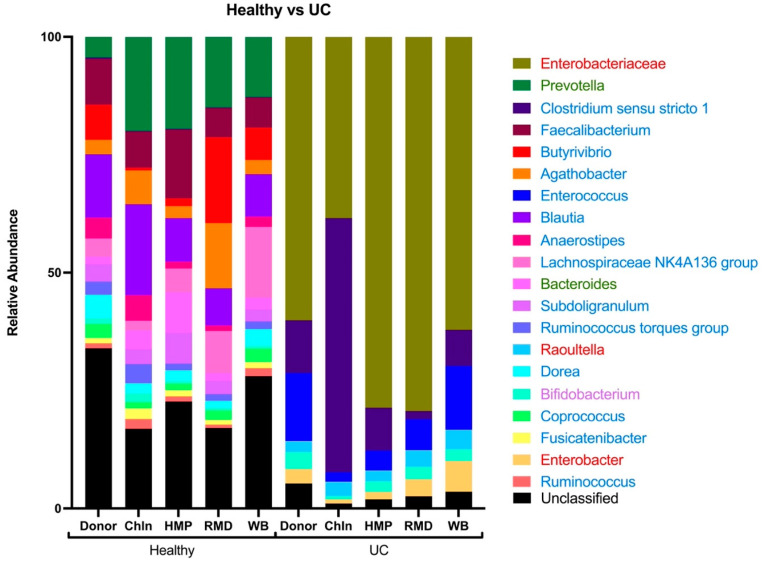
Taxonomic diversity plot showing the relative abundance of taxa at the genus level in each treatment group. Twenty of the most abundant taxa are displayed and sorted by different colors. Bacterial phylum is represented by different font colors, red font color belongs to *Proteobacteria*, green to *Bacteroidetes*, blue to *Firmicutes*, and purple to *Actinobacteria*.

A relative abundance of the twenty most abundant taxa was presented to assess the bacterial composition changes from the initial donor and the fiber treatments (Figure 9). A comparison of the two donor groups shows clear differences in the bacterial communities. UC, dominated by a few bacterial taxa groups (*Enterobacteriaceae*, *Clostridium sensu stricto 1* and *Enterococcus*, mostly), shows the definition of bacterial dysbiosis in a UC patient. The blooming of certain facultative anaerobes, such as those belonging to the *Enterobacteriaceae* family, and a decrease in the relative abundance of obligate anaerobes was observed. At the phylum level, we observed a decrease in the proportion of both *Firmicutes* and *Bacteroidetes*, while the proportion of *Proteobacteria* and *Actinobacteria* increased. Differences within the donor group and between fibers were observed. Within the healthy group, the fiber treatments showed indications of a higher relative abundance of *Prevotella* as compared to the donor. More noticeable, in the UC group, ChIn dramatically favored the increase in *Clostridium sensu stricto 1*.

## 4. Discussion

The physicochemical properties of dietary fibers play a significant role in their physiological effects and potential health benefits [26,27,28,29]. Although dietary fibers are known for their numerous health benefits, fiber consumption is low due to perceived intolerance by the American population [30]. Moreover, individuals with gut inflammation-related conditions such as UC are more sensitive to fiber intake. Along with physicochemical properties, bacterial dysbiosis associated with UC patients also plays a role in fiber intolerance [31,32,33,34,35,36]. The purpose of this study was to determine the role of microbial dysbiosis in the fermentation of fibers possessing distinct physicochemical properties.

A fast fiber fermentation rate was associated with higher total SCFA production (ChIn, AgIn, HMP and LMP) and higher gas production in a short period of time. High gas-producing fibers are known to exacerbate UC symptoms and may result in adverse effects [37]. In vitro fermentation demonstrated the existence of dietary fibers that do not produce much gas and have a moderate amount of total SCFA production, such as Chmuc and HACS. This indicates the possibility of selecting fibers that do not generate much discomfort due to gas, but still exert the health benefits due to SCFAs [38,39,40].

Within the physicochemical properties studied, the common notion is that solubility and fermentability go hand in hand. We observed that even though certain fibers were less soluble (oat bran, potato starch and the high amylose corn starch), their fermentation rates were similar to other soluble fibers. This suggests that solubility alone may not be a good indicator of fermentability, but this property can majorly be taken into consideration for its physical potential benefits in the gut, such as reducing diarrhea or constipation [41,42,43,44].

Bacterial dysbiosis is a common condition in UC patients and has been associated with its pathogenesis [45]. Even though a cause–effect relationship has not been elucidated, a decrease in microbial diversity is associated with this disease [31]. To assess the role of bacterial dysbiosis in the metabolism of the fibers in the gut, a fecal sample from a UC patient was used as an inoculum in a 24 h in vitro fermentation. pH was significantly higher for all the dietary fibers fermented with the UC feces in comparison to the healthy group with a similar bacterial load. Higher pH in the gut, despite fiber fermentation, may favor the growth of pathogenic bacteria and promote the low microbial diversity observed in a UC patient’s gut [46]. Gas production was significantly altered in the low diversity samples, in which all the fibers presented a lower amount of gas produced in the UC group. The fast-fermentable fibers (HMP and ChIn) presented significant differences from the earliest time point, while the medium–slow-fermentable fibers presented significant differences from the 12 h time point. The structure complexity of the fibers may also play an important role in the metabolism of these fibers in an environment with low bacterial diversity. When addressing total SCFA production, HMP—a highly fermentable fiber with a more complex structure—presented no significant differences at 6 and 12 h time points compared to ChIn, a less complex fiber. This suggests that the fermentation of ChIn was more sensitive to microbial dysbiosis and reduced diversity compared to HMP fermentation. HMP may be a more efficient fiber for a patient with bacterial dysbiosis in terms of the health benefits exerted by SCFA production compared to ChIn, disregarding the discomfort that its gas production may generate.

Fiber treatments resulted in a bacterial community that was significantly altered compared to the original UC donor community. Results may differ in an in vivo model, since SCFA are utilized for beneficial purposes such as improving the gut barrier function and reducing inflammation, which may then be translated into an increased bacterial diversity [47,48,49]. Bray–Curtis dissimilarity and the Weighted Unifrac analysis demonstrated different clustering patterns between the healthy and UC donor, concluding that the initial microbiome composition influences the potential role of fiber in modulating gut bacteria. As shown in the beta diversity analyses, dietary fiber treatments have a higher impact on shifting the bacterial community in low-diverse dysbiotic communities. The clustering of the fiber treatments within the healthy donor demonstrates that the fibers are not generating a significant change in their bacterial populations rapidly, indicating the stability of the gut microbiome. Additionally, it is important to note how the more fermentable fibers such as HMP, ChIn and RMD had a selective purifying pressure in the microbial community, resulting in a decrease in the alpha diversity. In contrast, WB treatment maintained the microbial diversity, indicating the lack of bacteria that are able to metabolize WB in the community. Overall, changes in the bacterial community by these fibers are highly impacted by the dysbiotic state in the UC donor. In the gut ecosystem, different bacteria have specialized mechanisms to access and ferment dietary fibers. Fibers like pectin and wheat bran possess more complex structures, including intricate polysaccharide matrices and branched configurations. *Bacteroides* species, for example, are adept at degrading these complex fibers due to their broad array of carbohydrate-active enzymes (CAZymes) that can break down diverse polysaccharide linkages [15]. On the other hand, fibers such as inulin and resistant maltodextrins have simpler, more linear structures. *Bifidobacteria* are particularly efficient at fermenting these less complex fibers, using specific enzymes like inulinases and glucosidases to hydrolyze the fructose and glucose polymers [15]. An important aspect of fiber degradation in the gut is cross-feeding, where one bacterial species breaks down a complex fiber into simpler molecules that other bacteria can then ferment. For example, primary degraders like *Bacteroides* can produce oligosaccharides and monosaccharides from complex fibers, which are then utilized by secondary fermenters such as *Faecalibacterium* and *Roseburia*, leading to a synergistic and diverse microbial ecosystem. In a dysbiotic state, where primary degraders are absent or present in very low abundance, the entire fermentation process can be disrupted. The absence/low abundance of key bacteria like *Bacteroides* or *Bifidobacteria* means that complex fibers may not be broken down efficiently, leading to reduced SCFA production. This imbalance can impair gut health, as SCFAs are vital for epithelial cell maintenance, immune modulation, and anti-inflammatory responses.

The relative abundances of the bacterial taxa clearly indicate gut bacterial dysbiosis in a UC patient [50]. Changes in the gut environment due to the disrupted gut barrier and a rise in nitrate production may explain the blooming of the Enterobacteriaceae family [51,52,53]. The *Enterobacteriaceae* family, being a facultative anaerobe, is favored by this shift in the gut environment, while this inhibits the growth and colonization of obligate anaerobes, hence the shift in the proportion of *Proteobacteria* and the decrease in the relative abundance of *Firmicutes* and *Bacteroidetes*.

The limitations of our study include our donor sample size. The results obtained from one UC patient may not be generalizable to all patients diagnosed with this disease. It is necessary to consider microbiome heterogeneity, and that the results shown in this study may be donor-dependent. Even within the same UC donor, factors such as disease state and severity can influence microbiota composition and generate different responses. However, these results clearly show the role of UC-associated gut bacterial dysbiosis in fiber fermentation. Future directions of this study will include more donors with distinct severity levels of UC and an in vivo mice study; both approaches will help us validate our results.

## 5. Conclusions

In summary, these findings demonstrated the role of dietary fiber’s physicochemical properties in fiber fermentation. These physicochemical properties are important in selecting a suitable fiber for UC patients to obtain potential health benefits with minimal intolerance. Additionally, gut bacterial dysbiosis has been shown to be highly influential in the metabolism of these fibers in the gut.

## Figures and Tables

**Table 1:** Dietary fibers used for in vitro experiments and their source.

Dietary Fiber	Source
Chicory Inulin (ChIn)	Sigma Aldrich I2255 (St. Louis, MO, USA)
Agave Inulin (AgIn)	Bought from local supermarket
High Methoxyl Pectin (HMP)	Sigma Aldrich 93854 (St. Louis, MO, USA)
Low Methoxyl Pectin (LMP)	Sigma Aldrich P9135 (St. Louis, MO, USA)
High Amylose Corn Starch (HACS)	Ingredion Hi-Maize 260 (Hamburg, Germany)
Resistant Maltodextrins (RMD)	ADM Fibersol-2 (Chicago, IL, USA)
Chia Mucilage (Chmuc)	Extracted [18]
Psyllium Husk (PSY)	Bought from local supermarket
Potato Starch (PST)	Sigma Aldrich S4251 (St. Louis, MO, USA)
Soluble Corn Arabinoxylans (SCA)	Extracted and donated by collaborating lab
Partially Hydrolyzed Guar Gum (PHGG)	Bought from local supermarket
Corn Bran (CB)	Bought from local supermarket
Oat Bran (OB)	Bought from local supermarket
Wheat Bran (WB)	Bought from local supermarket
Small-sized Cellulose (Scell)	Sigma Aldrich 310697 (St. Louis, MO, USA)
Medium-sized Cellulose (Mcell)	Sigma Aldrich C6288 (St. Louis, MO, USA)

**Table 2:** Soluble (SDF) and insoluble (IDF) fractions of the dietary fibers.

Dietary Fiber	SDF (%)	IDF (%)
LMP	89.54	10.46
HMP	81.39	18.61
SCA	72.31	27.69
PHGG	71.84	28.16
AgIn	71.29	28.71
Chmuc	70.13	29.87
RMD	66.82	33.18
PSY	65.83	34.17
ChIn	64.89	35.11
OB	37.01	62.99
HACS	28.74	71.26
PST	23.79	76.21
Mcell	16.19	83.81
Scell	8.68	91.32
WB	5.97	94.03
CB	5.65	94.35

**Table 3:** Fiber assignment based on in vitro fecal fermentation inoculated with healthy human fecal sample results.

Dietary Fiber	Total SCFA	24 h Gas Production	6 h Fermentation Rate	Scenario Classification
RMD	Medium	Medium	Medium	Scenario 1
PHGG	Medium	High	Medium	
SCA	Medium	High	Medium	
HMP	High	High	Fast	Scenario 2
LMP	High	High	Fast	
AgIn	High	High	Fast	
ChIn	High	High	Fast	
OB	High	Medium	Medium	
Chmuc	Medium	Medium	Slow	
CB	Low	Low	Slow	Scenario 3
Mcell	Low	Low	Slow	
Scell	Low	Low	Slow	
HACS	Low	Low	Medium	
WB	Low	Low	Slow	
PSY	Low	Low	Slow	

## Data Availability

The original contributions presented in the study are publicly available. Sequences were deposited in the NCBI sequence read archive (SRA) database under Bioproject PRJNA1063306.
